# Genetics Primer

**Published:** 2012

Genetics is the study of genes—the heritable information that contains the codes for proteins and other molecules which form and maintain an organism’s structure and function. In most organisms, these genes are found in strands of deoxyribonucleic acid (DNA) molecules. The specific structure of the DNA (described below) ensures that the genetic information can be passed from one generation to the next, while allowing for some reorganization that results in new variations and, ultimately, evolution.

Although nearly all cells in an organism have the same set of DNA (i.e., genome), the genomes vary among organisms, ensuring that (with few exceptions) each individual is unique. The degree of this variation is a measure of how closely related two organisms are. Thus, the differences among the genomes will be smaller among members of a family than among two completely unrelated persons, and those between related species (e.g., humans and chimpanzees) will be smaller than those between more diverse species (e.g., humans and flies).

Higher organisms are made up of various tissues and organs composed of cells with a range of functions, such as nerve, blood, or muscle cells. Yet all these cells contain the same genome. To achieve the necessary variation in cell structure and function, some DNA portions are “active,” or expressed, in certain cells and at particular developmental stages, leading to the production of different end products. In addition, the environment, to some extent, can influence gene expression, resulting in changes in how the organism functions in, and adapts to, its environment.

## What Is DNA?

DNA is a large complex molecule constructed from building blocks called nucleotides, each of which consists of a sugar molecule (deoxyribose) attached to an organic base. There are four organic bases and, accordingly, four different nucleotides called adenosine, cytosine, guanosine, and thymidine, generally referred to by their initials A, C, G, and T. In the cell, the nucleotides are arranged as long strings, with two strings interacting at the organic bases to form a double helix. Moreover, because of the chemical structures of these bases, their interactions are highly specific, so that T nucleotides in one strand only can interact with A nucleotides on the other strand and C nucleotides only can interact with G nucleotides. As a result, the two strands are said to be complementary. This feature is the basis for the ability of the DNA to be duplicated faithfully (at least for the most part) when cells divide so that all cells in an organism carry the same DNA sequence, which also can be passed on to the next generation. However, some variations or errors (i.e., mutations) can occur during this duplication, which lead to the variations that ensure the diversity of individuals within one species and also across species.

## How Is Genetic Information Converted Into Proteins?

The genetic information is encoded in the order of the nucleotides. A gene is a particular set of these nucleotides that serves as the blueprint for a specific protein. But how does the cell read this building instruction? When a protein is needed in the cell, the DNA double helix at the corresponding gene briefly splits into single strands. This allows certain proteins that mediate specific chemical reactions (i.e., enzymes) to copy the appropriate DNA strand by bringing in new nucleotides complementary to those in the strand (see figure). This process is called transcription. However, these new nucleotides contain a different sugar (i.e., ribose) and instead of the T nucleotides use a fifth nucleotide called uracil (U). The resulting new strand, which is made up of ribose-containing A, C, G, and U nucleotides, is called a ribonucleic acid (RNA). The RNA is released from the DNA (which then “zips” back up with its complementary strand) and is processed further into a messenger RNA (mRNA) that moves as a single strand out of the nucleus into the cytoplasm. There, other enzymes can bind to the mRNA and bring protein building blocks (i.e., amino acids) together to form chains. This process is known as translation (see figure). The order in which the amino acids are assembled is determined by the sequence of nucleotides in the mRNA, with blocks of three mRNA nucleotides representing one amino acid. The sequential steps of transcription and translation—from DNA through the intermediate mRNA to protein—are the process by which genes are expressed.

## Variations Among Genes

Variations among genes, known as polymorphisms, lead to the production of different gene products (i.e., proteins) and underlie the unique characteristics of each individual. In general, any given gene is quite similar to the same gene in another organism—in other words, the nucleotide sequence is conserved. For example, the genes that code for the alcohol-metabolizing enzyme alcohol dehydrogenase are of the same size and base sequence in most individuals. However, small differences in the base sequence, affecting as little as one nucleotide, can result in a different protein. In some cases, these modifications still allow the gene to produce a functional protein but with slight variations that may affect its function. Other changes in the nucleotide sequence, however, may result in a nonfunctional or incomplete protein. The ability to conserve the sequences coding for important enzymes clearly is important to cells and organisms.

One important aspect of current genetics research is the identification of groups within populations that carry polymorphisms in various genes, resulting in gene variants called alleles. Identifying these polymorphisms can help scientists to better understand how the functions of these genes and their encoded proteins differ and how they relate to certain diseases or other characteristics. For example, do some people produce a form of alcohol dehydrogenase that is more (or less) efficient than other people? And does this influence their risk of developing alcoholism?

To better understand how genes relate to diseases and other characteristics, the National Institutes of Health created the Human Genome Project, which set out to map the human genome by sequencing the entire DNA sequence found in human cells. (Similar mapping efforts also have been conducted for many other organisms.) This project determined not only that the DNA sequence is highly conserved among humans (about 99.5 percent is the same in all humans) but also that numerous combinations of genetic variants exist that are transmitted together and which are known as haplotypes.

To identify such variations in populations with large numbers of samples, researchers are using genetic probes for specific genes with known sequences. These probes typically are short DNA or RNA sequences complementary to a distinctive portion of the gene of interest. This probe then is marked with, for example, a fluorescent tag so that it can be detected if it interacts with the corresponding complementary DNA sequence in a sample.

This idea of using probes to identify differences in DNA sequences can be expanded into broad-scale studies analyzing numerous gene variants across a number of organisms. These genome-wide association studies (GWAS) allow researchers to identify large numbers of variants and to relate them to different outcomes—for example, those associated with different diseases, such as alcoholism.

Identifying candidate genes of particular significance, either through individual probes or large-scale methods such as GWAS, then allows more detailed study of the particular characteristics and expressions of those genes and their role in disease.

## Figures and Tables

**Figure:**
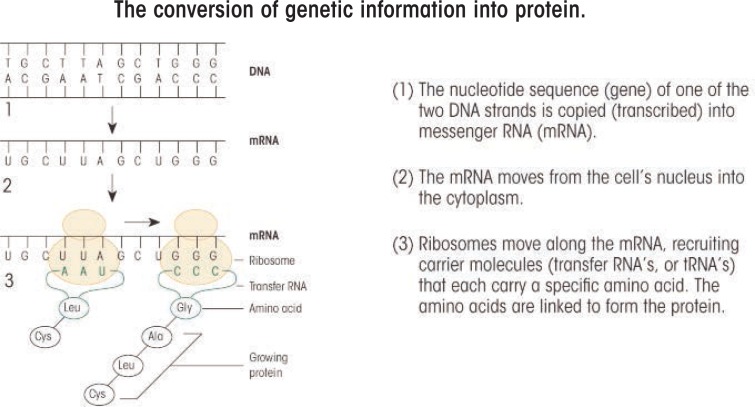
Steps 1–3a show the way in which the information on a DNA strand is transcribed into mRNA and then translated into proteins. SOURCE: Hiller-Sturmhöfel, S.; Bowers, B.J.; and Wehner, J.M. Genetic engineering in animal models. *Alcohol Health & Research World* 19(3):206–213, 1995.

